# The Preceptors’ Toolkit for Working with Struggling Pharmacy Students

**DOI:** 10.3390/pharmacy13030066

**Published:** 2025-05-02

**Authors:** Christina L. Mnatzaganian, Caitlin M. Gibson, Lisa Kipper, Charlene R. Williams, Tram B. Cat

**Affiliations:** 1 Skaggs School of Pharmacy and Pharmaceutical Sciences, University of California San Diego, La Jolla, CA 92024, USA; 2 School of Pharmacy, Virginia Commonwealth University, Richmond, VA 23284, USA; gibsoncm3@vcu.edu; 3 College of Pharmacy, Marshall B. Ketchum University, Fullerton, CA 92831, USA; lkipper@ketchum.edu; 4 Eshelman School of Pharmacy, University of North Carolina, Asheville, NC 28804, USA; 5 School of Pharmacy, University of California San Francisco, San Francisco, CA 94143, USA; tram.cat@ucsf.edu

**Keywords:** education, experiential education, preceptor, students, pharmacy

## Abstract

Pharmacy preceptors often feel unprepared to support and manage underperforming student pharmacists in experiential education settings. Further, there is little guidance on how preceptors can best support struggling student pharmacists with mental health concerns or those requiring disability accommodations. Further, recent literature has demonstrated elevated rates of preceptor burnout when working with difficult students. Resources to address challenging experiential student pharmacist situations were gathered through a literature review and from several offices of experiential education. This toolkit aims to provide strategies and resources to support preceptors working with struggling student pharmacists, particularly those facing mental health challenges and/or those requiring accommodations.

## 1. Introduction

Pharmacy preceptors are often challenged to assess and appropriately address suboptimal student pharmacist performance [1]. Preceptors have reported feeling unprepared to support and manage underperforming and failing student pharmacists in experiential education (EE) settings [2]. It may be especially daunting to serve as the “gatekeepers” between student pharmacists, who only need to complete Advanced Pharmacy Practice Experiences (APPEs) to fulfill graduation requirements, and the “real world”, where they are expected to function as independent pharmacy practitioners. It is critical to consider the implications of allowing students to progress when they are not appropriately prepared to practice independently [3]. Students who advance without demonstrating adequate competency may negatively impact patient safety, postgraduate training, and the pharmacy profession.

While the prevalence of challenging situations involving pharmacy students remains unexplored, the existing literature does highlight similar concerns among other healthcare trainees, for instance, up to 28% of medical students have been noted to have challenging experiential situations [4]. The COVID-19 pandemic has further intensified disruptions in pharmacy experiential settings [5]. Compounding this issue, elevated rates of pharmacy preceptor burnout have been observed among practitioners working with difficult or unmotivated learners [6].

There is abundant research on how to identify deficiencies, provide feedback to struggling students, and remediation efforts that may be implemented. Mohammad et al. recently added to this literature by providing strategies to help preceptors identify student challenges and a stepwise approach to facilitate student success on rotations [1]. However, there remains a paucity of data on how to manage struggling students who are not demonstrating minimum competency to pass rotations. Additionally, research is lacking on how to support struggling students with mental health challenges and/or requiring disability accommodations. In response to the concerns previously described, this toolkit aims to offer strategies and resources to support preceptors in working with struggling students, particularly those facing mental health challenges or requiring accommodations. The resources included were gathered through a literature review and supplemented by contributions from the authors’ respective offices of experiential education (OEE).

## 2. Identifying Deficiencies and Contributing Factors

### 2.1. Identifying Deficiencies

To respond appropriately to underperforming students, preceptors must be equipped with resources, tools, and strategies to assess student performance and identify deficiencies. Preceptors should familiarize themselves with the school’s EE program learning goals, objectives, competencies, evaluation scales, grading, and policies and procedures concerning performance deficits [7]. Additionally, preceptors must create a culture of safety, professionalism, and respect [7]. Preceptors should communicate clear expectations and determine learner goals and opportunities at the beginning of the rotation [7,8]. Finally, preceptors should be trained to identify various deficiencies and challenging learning situations, which may be exacerbated by Social Determinants of Learning™ (SDOL), to appropriately intervene and seek support [1,9].

Steinert proposed a model to analyze learners’ challenges from multiple angles, identifying both challenges and strengths [9]. Learner problems may be singular or could overlap with each other. Deficiencies may relate to: (1) the lack of knowledge of basic or clinical sciences, (2) attitudinal problems presenting as challenges with motivation, insight, self-assessment, and relationships with others on the team or patients, and/or (3) technical or clinical skill deficiencies or organization of work [9]. Challenges with clinical skills within pharmacy training may include difficulty in identifying medication related problems, data gathering and interpretation, prioritizing problems, assessing, and developing a care plan that includes treatment, monitoring, and education [10].

Once deficiencies are identified as either knowledge-, attitude-, and/or skills-based, preceptors should assess whether the issue(s) reside(s) at the preceptor, system, and/or learner level(s) [9]. Preceptors who are unsure of where to begin should contact the school’s OEE for assistance. Additionally, the Steinert framework tool may be used for organizing, classifying, and analyzing learner strengths and deficiencies as well as root causes (Appendix A, Table A1). Deficiencies should directly relate to learning outcomes and/or formal evaluation criteria. Mohammad et al. offer learning environment, SDOL, and medical conditions as additional factors that may impact the learner [1].

Preceptors should evaluate the deficiency based on a variety of considerations: whether the situation is isolated or ongoing, potential root causes, and what could make the situation better or worse [9]. Further, preceptors should determine students’ perception or self-awareness of the deficiency, strengths and areas needing improvement regarding knowledge, attitudes, and skills, the students’ SDOL, and the preceptor’s perceptions, feelings, and expectations as applicable [1,9]. If there is concern the student will not be successful at any point in the rotation, early collaboration with the OEE is essential to support both the preceptor and student.

### 2.2. Contributing Factors

In addition to life stressors (e.g., moving, financial troubles, housing insecurities, interpersonal conflicts), substance misuse, learning disabilities, or mental health challenges may further hinder students’ progression in their experiential training. Students with specific learning disabilities (SLDs), mental health challenges, and experiential accommodations have increasingly become topics of concern and discussion among OEEs.

While 94% of students with SLD receive accommodations in kindergarten through grade 12, only 17% of students receive accommodations in postsecondary education [11]. An even smaller percentage (0.7% to 3%) of graduate or professional students disclose their SLD to their academic institution [12]. Among pharmacy students receiving accommodations, support is more commonly provided in the classroom than in the experiential setting. However, limited guidance exists on best practices for supporting students with SLD and mental health challenges in experiential practice [12,13]. The most common challenge experiential administrators faced in addressing the needs of students with disabilities was students not disclosing their disability (58%), followed by lack of understanding from preceptors (25%) [13]. Unfortunately, while stigma around SLD is slowly dissipating with increased awareness across the academy, students may still hesitate to disclose their disability out of fear that preceptors will negatively bias them.

With mental health challenges and burnout on the rise, colleges and schools of pharmacy are determining how to best monitor and address student pharmacists’ wellness and mental health, recognizing these issues may contribute to poor student performance [14,15,16]. When examining the relationship between burnout and engagement, students with emotional exhaustion and professional inefficacy, such as lack of educational accomplishments, tend to have negative perceptions of their academic achievements, thereby making them more likely to be frustrated, perform poorly, and ultimately withdrawn from their responsibilities [17]. In fact, 39% of students’ academic dissatisfaction is related to burnout [18]. Faculty/preceptor support in the learning environment or personal life circumstances can also influence a learner’s wellbeing and motivation to learn [19,20].

In response to these concerns, the American Association of Colleges of Pharmacy has made student wellbeing one of its top strategic priorities [21]. Through increased efforts towards evaluating strategies to better monitor and promote student wellbeing, multiple intrinsic and extrinsic factors have been recognized as important drivers of student burnout. These factors include workload, learning environment culture and values, meaningful pharmacy school experiences, relationships, and personal factors [20]. Promotion of wellness strategies in pharmacy education varies by institution ranging from individual-level interventions, such as encouraging students to practice mindfulness and meditation during didactic coursework, to organization level interventions, such as training of EE faculty to know when and how to assist students in distress and implementing wellness programs for APPE students [22,23,24].

## 3. Early Intervention

Early intervention allows preceptors to address specific problems early on, thereby potentially decreasing chances of student failures. In early intervention, the school’s OEE can provide support and guidance to preceptors to monitor and set clear expectations with struggling students [25,26,27]. Primary and secondary strategies to circumvent difficult learning situations have been compiled as a checklist in Appendix B.

### 3.1. Primary Strategies

Strong rotations begin before the rotation start date with initial communication and expectations relayed to students, ideally weeks prior to the rotation start date. Preceptors may assign a pre-rotation survey to learn students’ strengths, areas of improvement, rotation goals, and career interests to better align expectations and tailor the learning experience to the student. Preceptors should incorporate a robust orientation on the first day that includes reviewing a structured learning description, mutual goal setting, early knowledge and skills assessments, setting rotation expectations, introduction to team members, and the appropriate application of the four preceptor roles [8,28,29]. To minimize the likelihood of students forgetting detailed expectations during the remainder of the rotation, preceptors may consider reminding students of expectations during regular feedback sessions and/or video recording expectations for students who are more visual learners. While primary strategies may minimize problems, some students will still struggle on rotation.

### 3.2. Secondary Strategies

Secondary strategies with resources should be distributed to students with deficits in knowledge, attitude, and/or skills as they arise [8,9]. When deficits are identified, preceptors should have open and honest face-to-face dialogue with the student and brief the OEE about the student’s ability to be successful on rotation, particularly if there is concern the student will not pass.

Preceptors may wish to consult with the OEE to request previous performance data on current students for insights on strengths and weaknesses. However, there may be regulations that govern the type of information that may be shared as well as who may access student data. For example, the Family Educational Rights and Privacy Act (FERPA) was enacted as federal law to protect the privacy of student education records [30]. Under FERPA, student records beyond name and dates of attendance (unless they have opted out of being listed in directory information) may not be released without student consent. Certain exceptions to this rule apply such as “school officials with legitimate educational interest”, however, how schools determine if preceptors are considered “school officials” is a gray area and data sharing may vary with different interpretations of the law [30]. Regardless of FERPA implications, OEEs should work with preceptors to implement individualized education plans (IEPs) for the student and to support the preceptor. Resources are available that describe teaching and remediation strategies and activities to overcome identified deficits [27,31].

Implementing secondary strategies to address performance concerns requires strong communication skills, formulating clear and concise plans, a comprehensive approach to feedback, and follow-up on student performance. Despite the frequency of feedback required in experiential learning, preceptors cite giving effective feedback as a high priority professional development need [32]. Guidance on providing feedback, having crucial conversations, documentation, and addressing learning disabilities and accommodations will be described below.

#### 3.2.1. Structure and Timing of Feedback

Regular scheduled feedback sessions should occur throughout the rotation (e.g., “Feedback Fridays” may be a useful framework to assess student perceptions of performance and invite collaborative discussions on strengths and areas for improvement). Regular scheduled feedback should be coupled with ongoing, just-in-time, verbal formative feedback that immediately follows routine rotation activities (e.g., patient care decisions, drug information lookups, topic discussions) to facilitate recall of specific examples of knowledge, skill, or attitude deficits. Just-in-time verbal feedback reinforces regularly scheduled formal feedback and allows students to connect specific examples with the preceptor’s assessment of their performance and is useful for real-time communication of performance [33]. Examples of effective feedback techniques include start-stop-continue and the one-minute preceptor technique [29,34]. Closed-loop communication about feedback involves ensuring the student understands the preceptor’s expectations moving forward through their ability to articulate and summarize the conversation.

Preceptors should also assess the most appropriate setting for constructive feedback. Generally, early discussions should occur privately between the preceptor and the student. If student deficits are severe, consistent, or outside of the comfort level of the preceptor, requesting joint feedback with the school’s experiential team is appropriate. While some students openly accept and easily translate feedback into improved performance, other students may be less able or willing to receive constructive feedback. Preceptors can increase the likelihood of effective conversations by cultivating a safe environment with the learner and defining the intention for the feedback (e.g., to help the student hone their knowledge, skills, and attitudes as a future pharmacist) [31].

Communication regarding deficiencies should be bi-directional. It is important for preceptors to understand how students self-appraise, and to work toward agreement in student and preceptor perceptions of performance. Various meta-cognitive frameworks can be used to facilitate this process. One common example is the Johari window, which can be used to facilitate discussions of self-awareness in experiential training [35]. The Johari window can organize feedback and help students identify blind spots by comparing student self-assessment with outward perceptions of student performance [36,37].

#### 3.2.2. Crucial Conversations

After establishing what is going well and what requires improvement within a rotation, preceptors should clearly communicate what this means for the student, including specifically stating if the student is on track to pass the rotation or not. Discussions around performance become “crucial conversations” when strong emotions, differing opinions, or high-stakes scenarios are present [38]. While avoiding such conversations may be tempting, ultimately the situation may snowball without prompt and direct attention.

Adequate preparation (e.g., rehearsing orally, outlining key points) is key to handling crucial conversations. In addition to preparing information that needs to be relayed to the learner and anticipating potential reactions, preceptors must also practice self-awareness. For example, acknowledging stress or vulnerabilities preceptors may be feeling prior to the discussion is critical to ensuring and delivering focused objective feedback [38,39]. Setting the stage with intentions for the conversation may also diffuse potentially heated discussions [38,39].

When providing constructive feedback, preceptors should clearly communicate the gap between observed and expected behaviors, citing specific examples as frequently as possible. It is important that the student understands how improving knowledge, skills, or attitudes could result in a better outcome. Furthermore, the learner should be assured that they are not a “bad student”, rather, there are specific learning and/or behavioral areas that must be improved. Should the student not be receptive, the preceptor should explain how dismissing feedback demonstrates a deficit in professionalism, and model how the student can respond to future feedback. The preceptor may further contact the OEE based on the nature of the student’s response for further guidance and support.

#### 3.2.3. Documentation

Documentation of feedback through emails, evaluation platforms, and/or IEPs is necessary as a secondary strategy. Students can refer to written examples as they work to improve performance, and preceptors have documented evidence of deficiencies if the student does not show progression. Furthermore, formal written feedback is useful for the OEE in devising IEPs and ensuring longitudinal success of struggling students. IEPs are also beneficial for students who are not progressing due to socio-behavioral issues such as professionalism deficits, communication issues, and lack of self-awareness [40].

OEEs should provide IEP templates to preceptors when needed and IEPs should be written to provide guidance that is specific, measurable, and time-bound. Preceptors, in collaboration with OEE, should clearly document and communicate measures of success criteria so the student understands what needs to be achieved for a passing grade. Additionally, the student should be involved in devising the plan to ensure understanding and to gain buy-in as well as improved self-awareness and lifelong learning skills [27,41]. Preceptors should review the IEP in detail with the student, clarifying any questions or confusion, and request that the student sign the document. The OEE should receive a copy of this plan once finalized and may further implement a contract between the student and the school. Finally, the preceptor should reassess the student’s performance on a regular, pre-defined basis to ensure continued improvement as described on the IEP. Appendix C, Table A2 provides a hypothetical IEP for a student demonstrating deficits in clinical skills.

#### 3.2.4. Addressing Disabilities and Accommodations

The management of disabilities could look different based on each SLD and the learning environment. When working with students with SLDs, there are some key principles to ensure that reasonable accommodations are provided in EE. Accommodations should be made based on a reliable diagnosis, and they should remove disability factors that could potentially confound the assessment of students’ technical ability to meet competency [12]. In addition, it is essential that communication and collaboration among the OEE, preceptors, students, and the Office of Student Disability (OSD) occurs to tailor students’ accommodations for each practice experience [12]. With these key principles in mind, the OSD should review the professional technical standards and work with the OEE and the practice site to help develop reasonable accommodations for a student’s learning experience. Spencer et al. noted that most approaches taken to accommodate students with learning disabilities on rotations include removing physical barriers, providing auditory/visual aids, simulation exercises, and removal of attitudinal barriers [13]. For students who have difficulty concentrating, a private quiet space for working up patients could be provided. In cases where students have anxiety or difficulty organizing tasks and need time to adjust and prepare for work, a schedule of topics or tasks to be completed in advance of the rotation could be provided. It is important to recognize, however, that certain accommodations do not always translate well to the experiential environment, posing challenges to preceptors and students alike. Therefore, open communication between the OEE, OSD, and practice site is essential for a supportive and successful student learning experience.

Sometimes students do not anticipate needing accommodations while on rotation and may consequently skip consulting with the school’s OSD. To help create a space and encourage students to feel comfortable disclosing their disabilities, a pre-rotation questionnaire that addresses students’ learning preferences and skill development goals is recommended. By doing this, preceptors can tailor their students’ learning experiences and set their students up for success. In cases where formal accommodations have not been sought or would be too untimely to do so during the rotation, preceptors on their own accord may decide to grant certain accommodations. However, preceptors are not legally required to do so without a school approved accommodation. Students should be encouraged by both preceptors and the OEE to seek necessary accommodations whenever possible to minimize barriers to their success.

There are several strategies that schools of pharmacy can use to improve the likelihood that students will seek out accommodations for their EE training and increase preceptor understanding. EE orientations at schools should include discussion of accessibility services and encourage students to meet with OSD representatives to discuss the need for accommodations. A reminder of the school’s technical standards should also be included during orientation and emailed regularly or posted in the school’s learning management software for both students and preceptors to review. Preceptors have reported a lack of resources and training on learning disabilities, uncertainty about implementing accommodations, and a need for professional development in this area [13,42]. As such, pharmacy schools should provide resources and training to preceptors in the form of continuing education regarding student learning disabilities and accommodations and/or one-on-one training based on individual student needs. Schools should collaborate with the OSD to establish protocols for students needing accommodations in the didactic curriculum, ensuring there is a seamless transition into experiential settings.

When it is unclear whether a poorly performing student on rotation is getting the appropriate accommodations for their SLD or has academic deficiencies, applying a formalized student remediation process could help uncover the underlying problem. It could also help guide future decisions making student success in experiential learning possible. Patwari et al. used a diagnostic objective structured clinical examination (OSCE) in a medical student with disabilities to determine the need for additional or refined accommodations versus clinical remediation as well as root causes for the poor performance [43]. Pharmacy students who participated in OSCEs prior to their first APPE described feeling more prepared and less stressed for their rotations [44]. There may be value in offering OSCEs during pharmacy APPEs as well. Preceptors who feel that students would benefit from extra time completing pharmacy-based simulations through an OSCE could work with the corresponding OEE to arrange this, when applicable.

## 4. Unsuccessful Early Interventions

Unfortunately, despite early interventions through primary and secondary strategies, not all students will attain the required learning outcomes to pass a given rotation. It is important for the preceptor to remember their role is to assign the most appropriate grade to the student based on their performance on the given rotation; preceptors should not allow concerns about overall implications of failing a rotation to influence their assessment. Preceptors experiencing consternation regarding student progression must remember that the student failed to achieve the rotation objectives. Ultimately, the OEE and school will holistically assess the student’s academic and professional performance, making appropriate decisions about tertiary strategies, which may range from extended time on the given rotation, repeating the rotation, extending time in the curriculum, to dismissing the trainee from the program per the school’s progression and dismissal policies [8].

## 5. Conclusions

Preceptors may find themselves challenged on how best to handle scenarios where students are not progressing appropriately. This toolkit serves to provide some helpful resources for preceptors to use and step by step approaches to address challenging learning situations while simultaneously working with OEEs. Preceptors are encouraged to work with students and schools closely on issues surrounding students’ mental health and accommodations as more data emerges on best practices.

## Data Availability

Not applicable.

## Abbreviations

The following abbreviations are used in this manuscript:

APPE	Advanced Pharmacy Practice Experience
EE	Experiential Education
IEP	Individualized Education Plan
OEE	Office of Experiential Education
OSCE	Objective Structured Clinical Exam
OSD	Office of Student Disabilities
SLD	Specific Learning Disability
SDL	Social Determinants of Learning™

## Appendix A

**Table A1:** Steinert framework for organizing and analyzing learner deficiencies applied to student pharmacists.

**Knowledge**	**Attitudes**	**Skill**
Example: Gaps in knowledge of foundational sciences, drug or clinical knowledge.	Example: Challenges with motivation, insight, self-assessment, pharmacist-patient relationships.	Example: Difficulties with interpreting subjective/objective information, interpersonal skills, technical skills such as physical assessment and calculations, clinical judgement, or organization of work and documentation.
**Preceptor**	**Student**	**System**
Example: Preceptor’s or team member’s perceptions, expectations, or feelings; personal experiences or stresses.	Example: Relevant life history or personal problems, including acute life stressors such as financial and relational, specific learning disabilities, psychiatric or other illness, or substance misuse; expectations and assumptions; response to feedback.	Example: Unclear rotation standards or responsibilities; overwhelming workload or inadequate staffing; inconsistent precepting or supervision; lack of ongoing feedback or assessment.

## Appendix B

A sample checklist for preceptors to use for struggling pharmacy students who are not progressing.

Student name:

School:

Rotation:

Preceptor name:

Date:

Primary strategies

☐ Student provided with rotation-specific learning description
☐ Reviewed learning description and expectations during orientation on first day
☐ Student provided with appropriate electronic medical record training
☐ Individual goals set with student

Secondary strategies

☐ Preceptor identification of student deficit(s) (select all that apply)
  ☐ Knowledge
  ☐ Skills
  ☐ Attitude/professionalism
☐ Provided feedback to student both verbally and in writing that identifies deficits
☐ Notified school experiential education team
☐ Created individualized education plan with student input and shared with experiential team
☐ Met with student at regular, pre-specified times to provide ongoing feedback, updated plan

If the student is not progressing per expectations delineated in written plan:

Tertiary strategies

☐ Student needs more time on this rotation; school may need to extend program
☐ Student needs to repeat this learning experience

## Appendix C

**Table A2:** Example of individualized education plan (set in an ambulatory care learning experience in a diabetes clinic) for preceptors to utilize in challenging learning situations.

Deficiency	Examples	Plan	Determinations of Success	Re-Evaluate
Skills: Patient file review	• Not reviewing patient’s primary care provider or other referring provider’s note to become familiar with the case. • Not reviewing labs, past medical history, vaccination status, and complications monitoring such as eye/foot exams. • Incomplete therapeutic plan not considering possible pharmacologic and non-pharmacologic treatments.	• Review patient’s file thoroughly and become fully familiar with patient’s chart before presenting the patient. • Determine suitable pharmacologic and non-pharmacologic treatments.	• Conduct through evaluation of patient and present pharmacologic and non-pharmacologic plan for at least 1 patient.	Weekly during Feedback Fridays
Skills: Patient encounter and interview	• Meeting with patients and not asking pertinent questions. • Unable to educate patients on hypoglycemia, how to use continuous or blood glucose monitor, how to use insulin and vial or insulin pen, and insulin dosing.	Conduct one full patient interview for at least 1 patient. 1. Subjective: use the questions listed in template and ask pertinent questions. 2. Medication reconciliation and educate patient on medications. 3. Educate on how to use insulin vial, syringe, expiration, storage, etc. 4. Educate patient on how to recognize and treat hypoglycemia. 5. Calculate correction scale and counsel on trend arrow scale. 6. Provide basic nutrition consultations.	• Conduct one patient interview in full scope, follow up, and educate patient on non-pharmacologic approach. • Educate patient on pharmacologic agents as needed. • Calculate insulin dose and educate patient.	Weekly during Feedback Fridays
